# Antibody seroconversion in asymptomatic and symptomatic patients infected with severe acute respiratory syndrome coronavirus 2 (SARS‐CoV‐2)

**DOI:** 10.1002/cti2.1182

**Published:** 2020-09-26

**Authors:** Chuanhao Jiang, Yali Wang, Min Hu, Lingjun Wen, Chuan Wen, Yang Wang, Weihong Zhu, Shi Tai, Zhongbiao Jiang, Kui Xiao, Nuno Rodrigues Faria, Erik De Clercq, Junmei Xu, Guangdi Li

**Affiliations:** ^1^ Department of Laboratory Medicine The Second Xiangya Hospital Central South University Changsha China; ^2^ Hunan Provincial Key Laboratory of Clinical Epidemiology School of Public Health Central South University Changsha China; ^3^ Department of Pediatrics The Second Xiangya Hospital Central South University Changsha China; ^4^ Department of Social Affairs The Second Xiangya Hospital Central South University Changsha China; ^5^ Department of Orthopedic Surgery The Second Xiangya Hospital Central South University Changsha China; ^6^ Department of Cardiology The Second Xiangya Hospital Central South University Changsha China; ^7^ Department of Radiology The Second Xiangya Hospital Central South University Changsha China; ^8^ Department of Pulmonary and Critical Care Medicine The Second Xiangya Hospital Central South University Changsha China; ^9^ Department of Zoology University of Oxford Oxford UK; ^10^ Department of Infectious Disease Epidemiology School of Public Health Imperial College London London UK; ^11^ Department of Microbiology, Immunology and Transplantation Rega Institute for Medical Research KU Leuven Leuven Belgium; ^12^ Department of Anesthesiology The Second Xiangya Hospital Central South University Changsha China

**Keywords:** COVID‐19, IgG, IgM, immune responses, SARS‐CoV‐2

## Abstract

**Objectives:**

Asymptomatic and symptomatic patients may transmit severe acute respiratory syndrome coronavirus 2 (SARS‐CoV‐2), but their clinical features and immune responses remain largely unclear. We aimed to characterise the clinical features and immune responses of asymptomatic and symptomatic patients infected with SARS‐CoV‐2.

**Methods:**

We collected clinical, laboratory and epidemiological records of patients hospitalised in a coronavirus field hospital in Wuhan. We performed qualitative detection of anti‐SARS‐CoV‐2 immunoglobulin M (IgM) and immunoglobulin G (IgG) using archived blood samples.

**Results:**

Of 214 patients with SARS‐CoV‐2, 26 (12%) were asymptomatic at hospital admission and during hospitalisation. Most asymptomatic patients were ≤ 60 years (96%) and females (65%) and had few comorbidities (< 16%). Serum levels of white and red blood cells were higher in asymptomatic than in symptomatic patients (*P*‐values < 0.05). During hospitalisation, IgG seroconversion was commonly observed in both asymptomatic and symptomatic patients (85% versus 94%, *P*‐value = 0.07); in contrast, IgM seroconversion was less common in asymptomatic than in symptomatic patients (31% versus 74%, *P*‐value < 0.001). The median time from the first virus‐positive screening to IgG or IgM seroconversion was significantly shorter in asymptomatic than in symptomatic patients (median: 7 versus 14 days, *P*‐value < 0.01). Furthermore, IgG/IgM seroconversion rates increased concomitantly with the clearance of SARS‐CoV‐2 in both asymptomatic and symptomatic patients. At the time of virus clearance, IgG/IgM titres and plasma neutralisation capacity were significantly lower in recovered asymptomatic than in recovered symptomatic patients (*P*‐values < 0.01).

**Conclusion:**

Asymptomatic and symptomatic patients exhibited different kinetics of IgG/IgM responses to SARS‐CoV‐2. Asymptomatic patients may transmit SARS‐CoV‐2, highlighting the importance of early diagnosis and treatment.

## Graphical abstract

We studied a cohort of 26 asymptomatic and 188 symptomatic patients in a coronavirus field hospital. We found asymptomatic cases, mostly young females ≤ 60 years, in approximately 12% of nonseverely ill cases. More than 90% of cases experienced immunoglobulin M (IgM)/immunoglobulin G (IgG) seroconversion at the time of virus clearance, whereas the median time from the first virus‐positive tests to IgG/IgM seroconversion was significantly shorter in asymptomatic than in symptomatic patients. Compared with symptomatic patients, asymptomatic patients had lower IgG/IgM titres and plasma neutralisation capacity at the time of virus clearance.

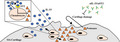

## Introduction

As of 28 August 2020, more than 24 million people have been infected with severe acute respiratory syndrome coronavirus 2 (SARS‐CoV‐2), and antiviral agents and vaccines are still under development.^1^, ^2^, ^3^ Although many strategies have been proposed to control and treat symptomatic patients with COVID‐19, early prevention of human‐to‐human transmission by asymptomatic patients remains a challenge. Moreover, asymptomatic patients carry SARS‐CoV‐2 with a strong transmission potential,^4^ but they are not routinely tested, especially in resource‐limited regions. In addition, several studies have reported asymptomatic patients in small cohorts.^4^, ^5^, ^6^, ^7^, ^8^, ^9^, ^10^, ^11^, ^12^, ^13^, ^14^, ^15^, ^16^ For instance, 13 of 23 SARS‐CoV‐2 cases in Washington state were asymptomatic at the time of viral screening.^5^ Approximately 40–45% of SARS‐CoV‐2 cases remain asymptomatic.^17^ Despite their importance in public health control, the serological and clinical features of asymptomatic carriers remain poorly understood.

Immunoglobulin G (IgG) and immunoglobulin M (IgM) are known antibodies for monitoring humoral immune responses to infections by viruses such as SARS‐CoV‐2.^18^, ^19^, ^20^, ^21^, ^22^, ^23^, ^24^ Because of their convenience and cost‐efficiency, new serological assays of IgG and IgM antibodies have been recognised as a promising diagnostic tool to complement viral nucleic acid screening in the at‐risk populations.^25^ Of note, IgG/IgM seroconversion can be observed in many patients after their infections with SARS‐CoV‐2,^4^, ^18^, ^20^, ^23^, ^26^ and serological responses are associated with disease severity.^19^ For instance, an observational study of 32 critically ill and 141 noncritically ill patients reported IgM and IgG seroconversion rates of 82.7% and 64.7%, respectively.^18^ Furthermore, IgG/IgM seroconversion could be detected along with a steady decline in viral loads in mildly ill patients,^4^ though IgG seroconversion can be observed at the same time or earlier than IgM seroconversion in symptomatic patients.^20^ Overall, IgM and IgG antibodies are useful biomarkers for monitoring disease progression in COVID‐19.^23^

Despite the above findings, the immune responses of IgG and IgM antibodies in asymptomatic patients remain poorly understood. This study aimed to characterise the serological and clinical features of asymptomatic and symptomatic patients from a field hospital in Wuhan that was temporarily established to treat nonseverely ill patients infected with SARS‐CoV‐2.

## Results

### Demographic and clinical features of asymptomatic and symptomatic patients

**Figure 1:**
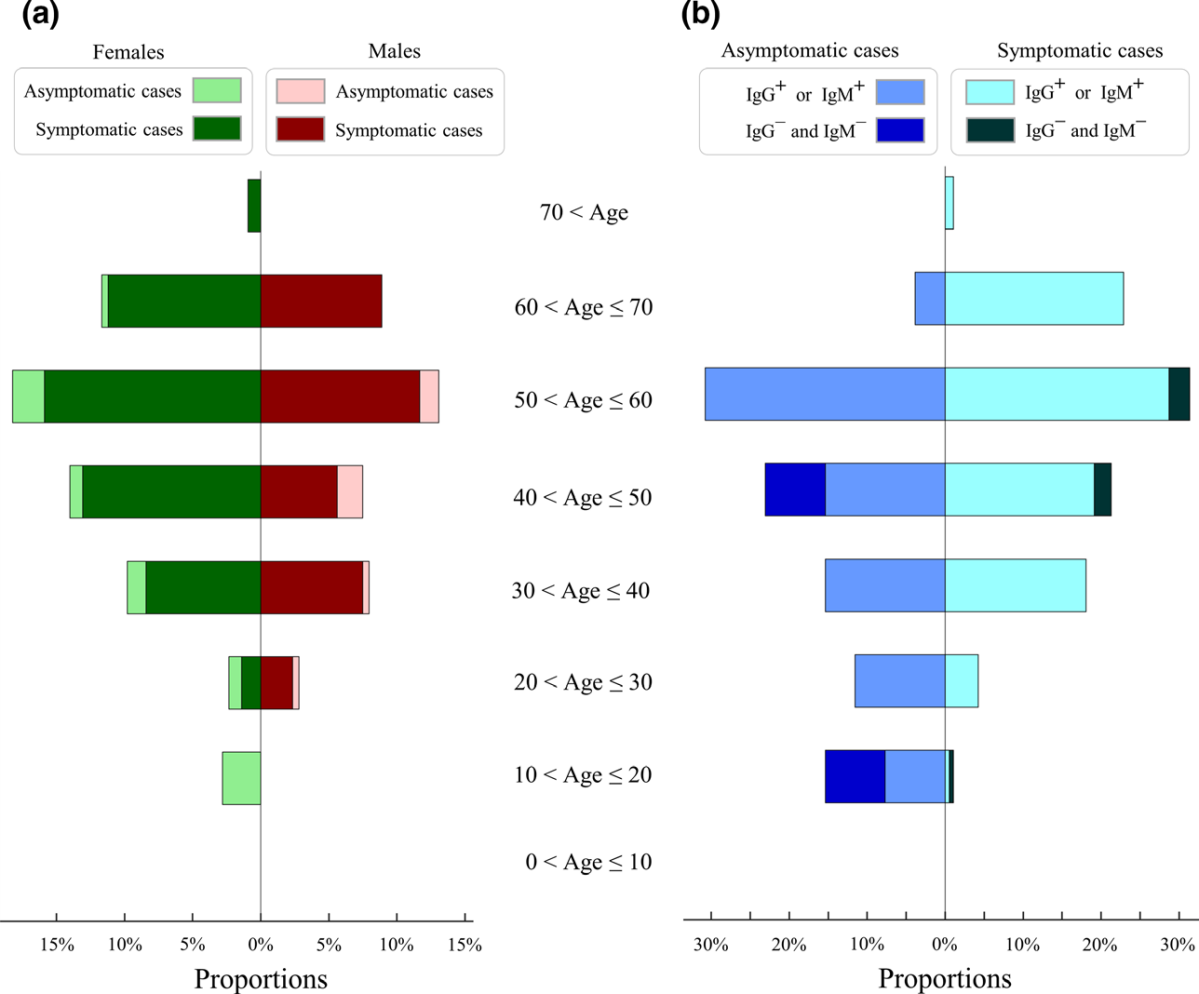
Demographic profiles of severe acute respiratory syndrome coronavirus 2 cases in different age categories. **(a)** Proportions of females (left) and males (right) in each age class. Proportions of asymptomatic and symptomatic females are shown by light‐green and dark‐green bars, respectively. Proportions of asymptomatic and symptomatic males are shown in light red and dark red, respectively. **(b)** Proportions of asymptomatic (left) and symptomatic (right) cases in each age class. Proportions of IgG^+^ or IgM^+^ seroconversion in asymptomatic and symptomatic cases are shown by light blue and light cyan, respectively. Proportions of IgG^−^ and IgM^−^ in asymptomatic and symptomatic cases are shown by dark blue and dark black, respectively. Results of IgG and/or IgM seroconversion were monitored by the time of hospital closure on 10 March 2020.

This study included a cohort of 214 nonseverely ill patients hospitalised in a field hospital in Wuhan between 5 February and 10 March 2020. Their clinical features are summarised in Table 1. Of the 214 patients infected with SARS‐CoV‐2, 128 (60%) were females and 168 (79%) were ≤ 60 years (median: 51, ranges: 11–82), as illustrated in Figure 1a. At hospital admission, the most common signs or symptoms included fever (67%), cough (66%) and fatigue (21%). Unlike the high proportions of comorbidities in severely ill patients,^27^, ^28^ only 20% of the 214 nonseverely ill patients had comorbidities such as hypertension (10%), diabetes (5%) or other diseases (5%).

**Table 1:** Baseline features of asymptomatic and symptomatic patients with COVID‐19

	Total (*N* = 214)	Asymptomatic (*N* = 26)	Symptomatic (*N* = 188)	*P*‐value
Age, median (IQR)^a^	51 (39–59)	42 (31–51)	52 (41–60)	0.001
≤ 60 years	168 (79%)	25 (96%)	143 (76%)	0.019
> 60 years	46 (21%)	1 (4%)	45 (24%)	
Gender				
Male	86 (40%)	9 (35%)	77 (40%)	0.54
Female	128 (60%)	17 (65%)	111 (60%)	
Signs or symptoms				
Body temperature	38.1 (36.7–38.4)	36.5 (36.4–36.7)	38.1 (37.8–38.5)	<0.001
Any symptom	188 (88%)	0 (0%)	188 (100%)	<0.001
Fever	144 (67%)	0 (0%)	144 (77%)	<0.001
Cough	141 (66%)	0 (0%)	141 (75%)	<0.001
Fatigue	45 (21%)	0 (0%)	45 (24%)	0.005
Chest tightness	23 (11%)	0 (0%)	23 (12%)	0.12
Myalgia	10 (5%)	0 (0%)	10 (5%)	0.48
Diarrhoea	9 (4%)	0 (0%)	9 (5%)	0.54
Headache	8 (4%)	0 (0%)	8 (4%)	0.60
Dyspnoea	7 (3%)	0 (0%)	7 (4%)	1.0
Palpitations	6 (3%)	0 (0%)	6 (3%)	1.0
Chills	5 (2%)	0 (0%)	5 (3%)	1.0
Comorbidities				
Any	42 (20%)	4 (15%)	38 (20%)	0.43
Hypertension	21 (10%)	2 (8%)	19 (10%)	0.86
Diabetes	11 (5%)	1 (4%)	10 (5%)	1.0
Cardiovascular diseases	7 (3%)	0 (0%)	7 (4%)	0.60
Chronic pulmonary disease	7 (3%)	1 (4%)	6 (3%)	1.0
Cancers	6 (3%)	1 (4%)	5 (3%)	0.57
Gastrointestinal diseases	3 (1%)	0 (0.0)	3 (2%)	1.0
Mental diseases	2 (1%)	0 (0.0)	2 (1%)	1.0
Laboratory biomarkers^b^				
White blood cells (×10⁹ L^−1^)	5.35 (4.67–6.40)	6.10 (5.06–6.58)	5.23 (4.57–6.37)	0.027
Lymphocytes (×10⁹ L^−1^)	1.59 (1.27–1.91)	1.86 (1.49–2.09)	1.52 (1.26–1.87)	0.017
Eosinophils (×10⁹ L^−1^)	0.08 (0.05–0.15)	0.13 (0.07–0.22)	0.07 (0.05–0.14)	0.02
Neutrophils (×10⁹ L^−1^)	3.29 (2.62–4.05)	3.50 (2.82–4.13)	3.22 (2.61–4.02)	0.41
Monocytes (×10⁹ L^−1^)	0.34 (0.29–0.43)	0.36 (0.30–0.43)	0.34 (0.29–0.43)	0.49
Basophils (×10⁹ L^−1^)	0.01 (0.01–0.02)	0.01 (0.01–0.02)	0.01 (0.01–0.02)	0.10
Red blood cells (×10^12^ L^−1^)	4.44 (4.08–4.77)	4.60 (4.46–4.83)	4.36 (4.04–4.73)	0.029
Haemoglobin (g L^−1^)	136 (128–145)	138 (132–146)	135 (126–145)	0.33
Platelets (×10⁹ L^−1^)	232 (195–279)	221 (205–270)	237 (194–281)	0.79
C‐reactive protein (mg L^−1^)	1.23 (0.45–4.01)	0.84 (0.22–1.87)	1.38 (0.49–5.28)	0.06

^a^ Interquartile ranges (IQR) of continuous variables are shown in the table.

^b^ Results were measured by the first biomarker tests within the first‐week hospitalisation. Normal ranges of biomarkers are listed in Supplementary table 1.

Of the 214 COVID‐19 patients enrolled in our cohort, 35 showed no symptoms at hospital admission, with nine of them developing mild or atypical symptoms during hospitalisation. In total, 26 (12%) asymptomatic patients exhibited no clinical symptoms at hospital admission or during hospitalisation; the others were symptomatic or presymptomatic (Supplementary figure 1). To characterise their clinical features, 26 and 188 patients were classified into asymptomatic and symptomatic groups, respectively (Table 1).

The asymptomatic patients were mostly young females (*N* = 17, 65%), and seven (27%) cases had family members infected with SARS‐CoV‐2. The asymptomatic group was significantly younger than the symptomatic group (median: 42 versus 52 years, *P*‐value = 0.001, Supplementary figure 2). Additionally, patients older than 60 years were less common in the asymptomatic group than in the symptomatic group (4% versus 24%, *P*‐value = 0.019). However, no difference between asymptomatic and symptomatic patients was found regarding the proportions of males and comorbidities (*P*‐values > 0.05, Table 1). Serum levels of white blood cells, lymphocytes, eosinophils and red blood cells at baseline were significantly higher in asymptomatic than in symptomatic patients (*P*‐values < 0.05, Table 1), and these biomarkers remained higher (but not significant) in asymptomatic patients during the second and third weeks of hospitalisation (Supplementary figure 3).

### IgG and IgM seroconversion

To monitor humoral immune responses of IgM and IgG antibodies, we tested all archived serum samples (*N* = 448) of the 214 patients during their hospitalisation. For each patient, IgG and IgM levels were measured using four archived serum samples (*N* = 18 patients), three serum samples (*N* = 61), two serum samples (*N* = 58) and one serum sample (*N* = 77). IgG/IgM antibodies were not assessed at the first virus‐positive screening because they were not required as hospitalisation criteria and blood samples were not collected before hospitalisation (see Methods).

First, IgG/IgM seroconversion was observed in both asymptomatic and symptomatic patients. Age distribution of IgG/IgM seroconversion was similar between asymptomatic and symptomatic patients (Figure 1b), while incidences of IgM or IgG seroconversion were also similar in males versus females and young versus old patients (*P*‐values > 0.05, Table 2). By the time of hospital closure, incidences of IgM and IgG seroconversions were observed in 147 (69%) and 199 (93%) patients, respectively (Table 2). Of 161 discharged patients with virus clearance, 149 (93%) had IgM or IgG levels ≥ 10 AU mL^−1^, and 156 (97%) had IgM or IgG levels ≥ 3 AU mL^−1^. During hospitalisation, two symptomatic cases experienced isotype switching from IgM ≥ 10 AU mL^−1^ plus IgG < 10 AU mL^−1^ to IgM < 10 AU mL^−1^ plus IgG ≥ 10 AU mL^−1^. IgG seroconversion was commonly found in both asymptomatic and symptomatic patients (85% versus 94%, *P*‐value = 0.07), whereas IgM seroconversion was less common in asymptomatic than in symptomatic patients (31% versus 74%, *P*‐value < 0.001, Table 3).

**Table 2:** Incidences of immunoglobulin G (IgG) and/or immunoglobulin M (IgM) seroconversion by the time of hospital closure

	IgG^+^, IgM^+^	IgG^+^, IgM^−^	IgG^−^, IgM^+^	IgG^−^, IgM^−^	*P*‐value
Asymptomatic (*N* = 26)	8 (31%)	14 (54%)	0 (0%)	4 (15%)	0.0002
Symptomatic (*N* = 188)	138 (73%)	39 (21%)	1 (1%)	10 (5%)	
Age ≤ 60 (*N* = 168)	114 (68%)	39 (23%)	1 (1%)	14 (8%)	0.18
Age > 60 (*N* = 46)	32 (70%)	14 (30%)	0 (0%)	0 (0%)	
Male (*N* = 86)	60 (70%)	21 (24%)	0 (0%)	5 (6%)	0.84
Female (*N* = 128)	86 (67%)	32 (25%)	1 (1%)	9 (7%)	

+: IgG or IgM ≥ 10 AU mL^−1^; −: IgG or IgM < 10 AU mL^−1^.

**Table 3:** Clinical outcomes of asymptomatic and symptomatic patients by the time of hospital closure

	Total (*N* = 214)	Asymptomatic (*N* = 26)	Symptomatic (*N* = 188)	*P*‐value
Hospital discharge	161 (75%)	20 (77%)	141 (75%)	0.83
Transferred to another hospital	53 (25%)	6 (23%)	47 (25%)	
Length of hospital stay (days)	17 (13–21)	13 (12–15)	18 (14–22)	0.001
Virus clearance				
SARS‐CoV‐2 clearance	180 (84%)	20 (77%)	160 (85%)	0.28
Time from FVS to virus clearance (days)^a^	16 (10–20)	10 (8–12)	16 (12–21)	<0.001
Symptom onset to virus clearance (days)	–	–	26 (20–33)	–
IgG				
IgG seroconversion	199 (93%)	22 (85%)	177 (94%)	0.07
Time from FVS to IgG seroconversion (days)	14 (8–17)	7 (5–8)	14 (9–19)	<0.001
Symptom onset to IgG seroconversion (days)	–	–	24 (18–29)	–
IgM				
IgM seroconversion	147 (69%)	8 (31%)	139 (74%)	<0.001
Time from FVS to IgM seroconversion (days)	14 (8–17)	8 (7–9)	14 (10–18)	0.001
Symptom onset to IgM seroconversion (days)	–	–	23 (18–29)	–

FVS: the first virus‐positive screening; IgG, immunoglobulin G; IgM, immunoglobulin M; SARS‐CoV‐2, severe acute respiratory syndrome coronavirus 2.

^a^ Interquartile ranges of continuous variables are shown in the table.

**Figure 2:**
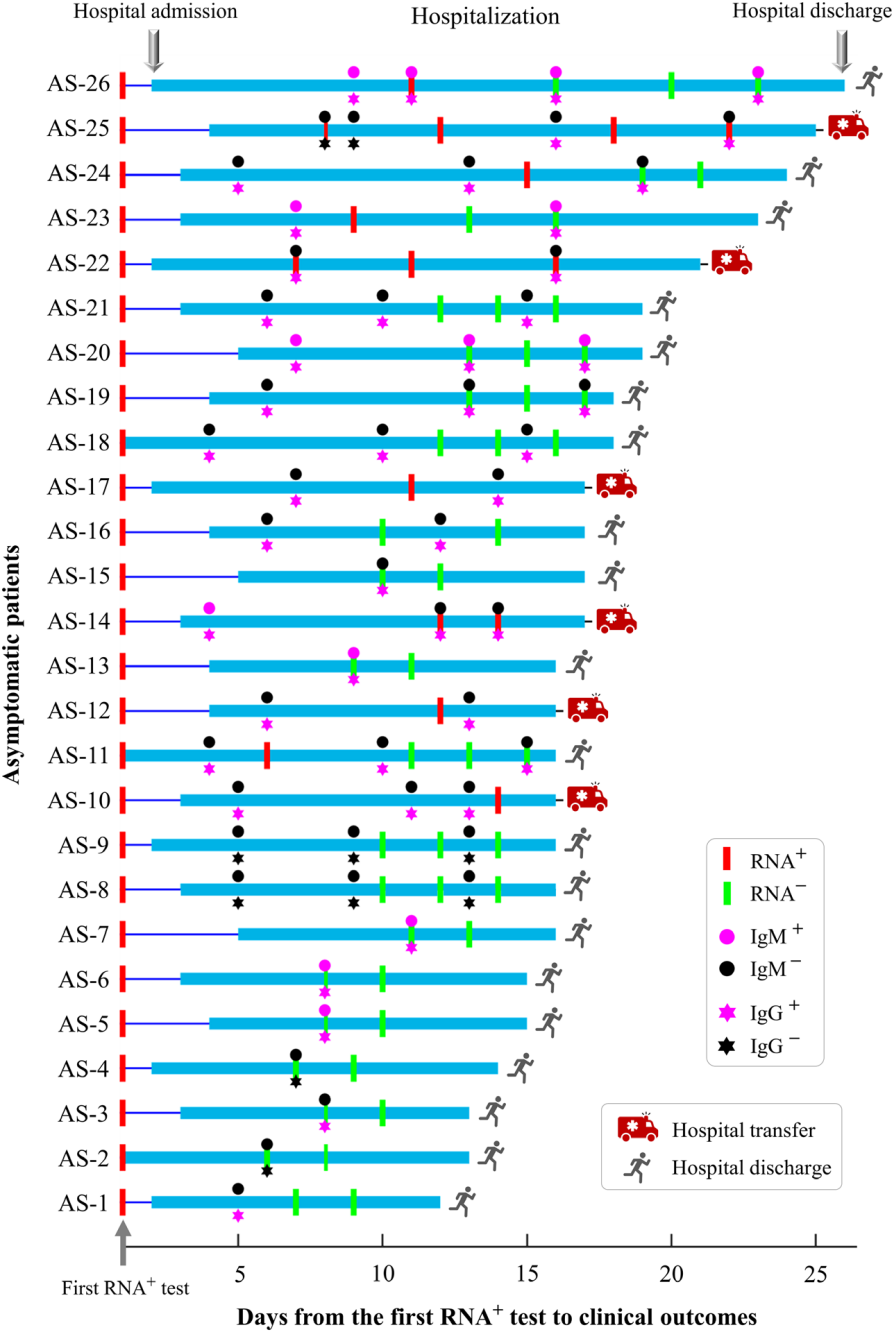
Timeline of viral RNA tests, immunoglobulin G (IgG)/immunoglobulin M (IgM) tests, hospital admission and hospital discharge in 26 asymptomatic patients.

Second, the median time from the first virus‐positive screening to IgG or IgM seroconversion was significantly shorter in asymptomatic than in symptomatic patients (median: 7 versus 14 days, *P*‐value < 0.01, Table 3). For 22 asymptomatic patients with IgG or IgM seroconversion, seroconversion was observed within 7 days in 14 (64%) patients, within 14 days in 21 (95%) patients and within 16 days in 22 (100%) patients (Figure 2). The earliest IgG/IgM seroconversion in asymptomatic patients was observed at approximately 4 days after the first virus‐positive test (Figure 2); for symptomatic patients, the median time from symptom onset to IgG/IgM seroconversion was approximately 23 days (Table 3).

**Figure 3:**
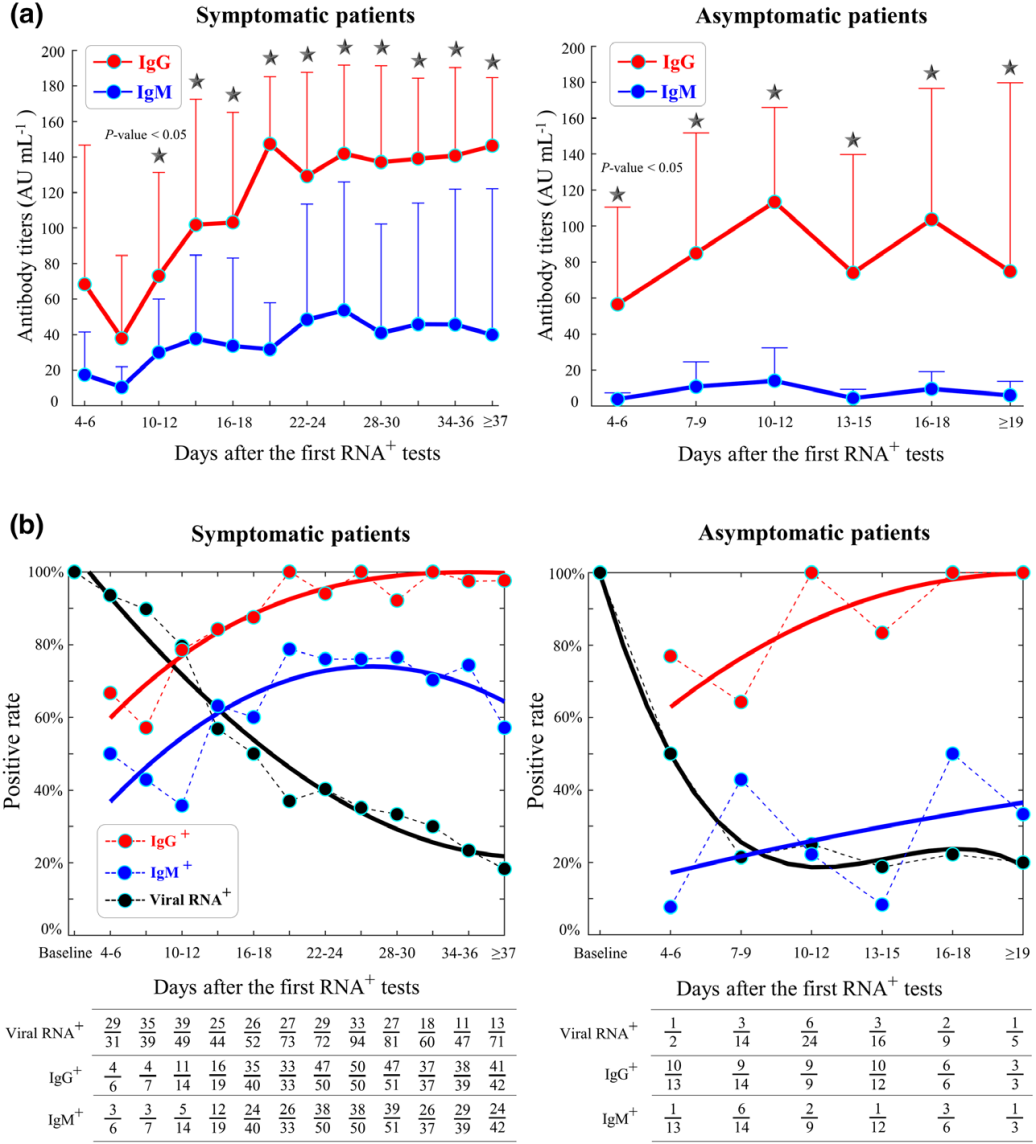
Dynamics of immunoglobulin G (IgG)/immunoglobulin M (IgM) and viral RNA during disease progression. **(a)** Mean and standard deviations of IgG and IgM titres in symptomatic (left) and asymptomatic (right) patients. A black star indicates a significant difference between IgG and IgM titres (*P*‐value < 0.05) at a sampling period of every three days. According to our clinical practice, serum samples at the first virus‐positive screening were not collected. **(b)** Positive rates of IgG seroconversion (red), IgM seroconversion (blue) and virus‐positive cases (black) in symptomatic patients (left) and asymptomatic patients (right). The table summarises the number of tested samples and positive results every 3 days. The polynomial fitting of positive rates is shown by the coloured curves. The *x*‐axis indicates the timeline from the first virus‐positive screening to the time point of IgG/IgM tests or viral RNA tests. Positive rates in the asymptomatic group were variable because of the small patient cohort (*N* = 26).

Third, the dynamics of average IgG and IgM titres were monitored at three‐day time points after the first viral RNA^+^ tests. For both symptomatic and asymptomatic patients, the increase in average IgG and IgM titres was mainly observed during the first 2 weeks (Figure 3a). IgG titres in asymptomatic patients were significantly higher than IgM titres at each sampling time point from 4–6 to ≥ 19 days (*P*‐values < 0.05). Such significance was also observed for symptomatic patients from 10–12 to ≥ 37 days (Figure 3a).

### IgG/IgM seroconversion emerging with the disappearance of SARS‐CoV‐2

During hospitalisation, IgG and IgM seroconversion occurred coincidentally with the decreasing number of virus‐positive cases confirmed by viral RNA tests (Figure 3b). IgG seroconversion rates in asymptomatic and symptomatic patients almost reached 100% at approximately 16–18 and 31–33 days, respectively. Furthermore, IgM seroconversion rates reached 76% at approximately 22–24 days in symptomatic patients, whereas relatively lower rates (≤ 50%) of IgM seroconversion were observed in asymptomatic patients at all sampling time points. By the time of hospital closure, a lower rate of IgM seroconversion was also observed in asymptomatic than in symptomatic patients (31% versus 74%, *P*‐value < 0.01, Table 3).

**Figure 4:**
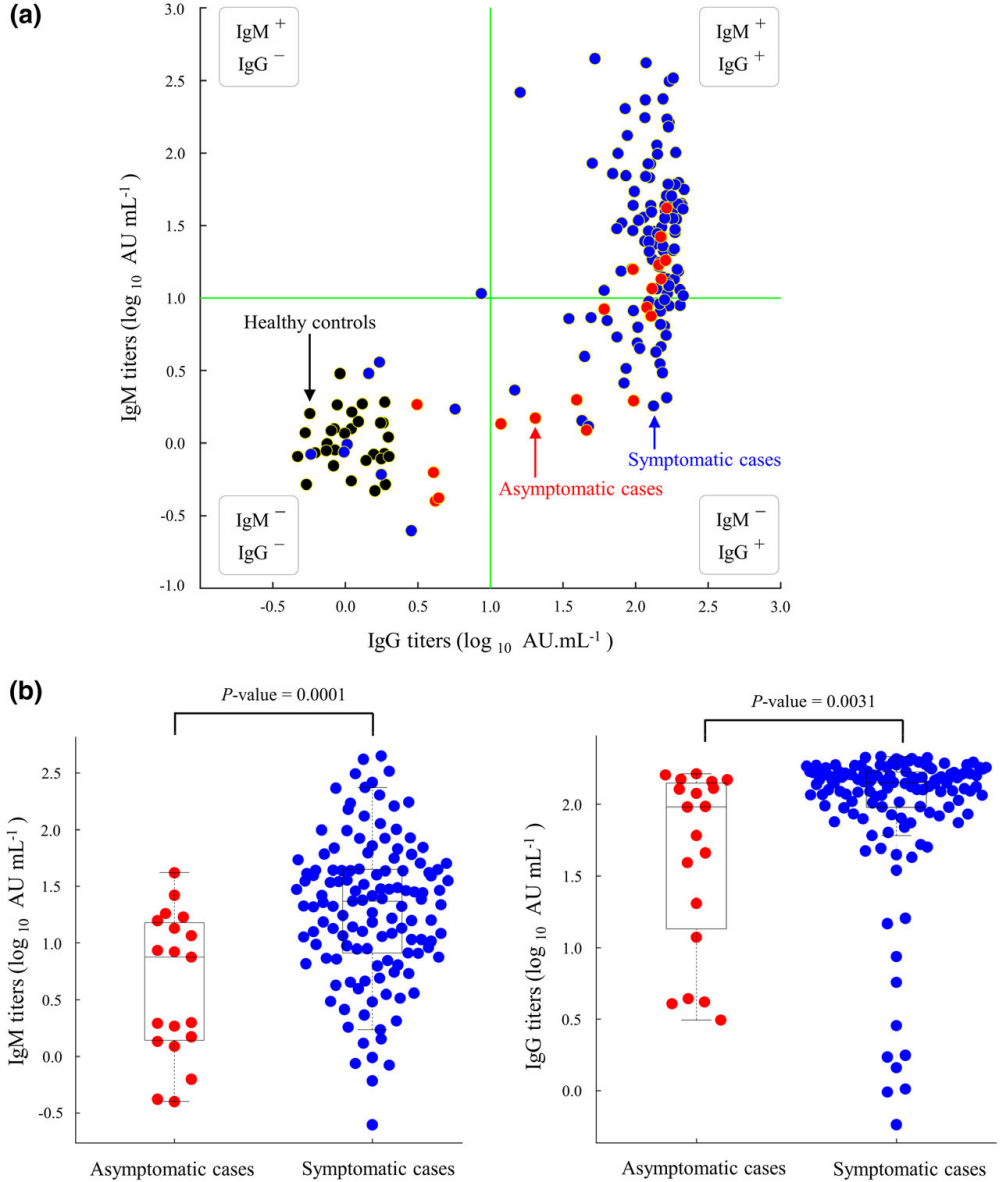
Plots of immunoglobulin G (IgG) and immunoglobulin M (IgM) titres measured at the time points of virus clearance. **(a)** Scatter plots of IgG and IgM titres in asymptomatic cases (red, *N* = 26), symptomatic cases (blue, *N* = 188) and healthy controls (black, *N* = 30). Green lines indicate cut‐offs of IgG and IgM at 1 log_10_ AU mL^−1^. IgG/IgM titres were measured at the time of virus clearance. **(b)** Box plots of IgM (left) and IgG (right) titres in asymptomatic and symptomatic patients.

Blood samples from 148 recovered patients were archived at the time of virus clearance. Of these 148 patients, only 12 (8%) showed no IgM or IgG seroconversion (Figure 4a). Compared with symptomatic patients, asymptomatic patients had lower titres of IgM antibodies (median: 0.87 versus 1.37 log_10_ AU mL^−1^, *P*‐value < 0.01) and IgG antibodies (median: 1.98 versus 2.15 log_10_ AU mL^−1^, *P*‐value = 0.003), as illustrated in Figure 4b. Further analysis confirmed that the average IgG and IgM titres of all sampling time points were significantly higher in symptomatic than in asymptomatic patients (*P*‐values < 0.01, Supplementary figure 4). Nevertheless, no difference in IgG or IgM titres was detected between young and old patients or between male and female patients (*P*‐values > 0.05, Supplementary figure 5).

### Neutralising capacity of patient plasmas

**Figure 5:**
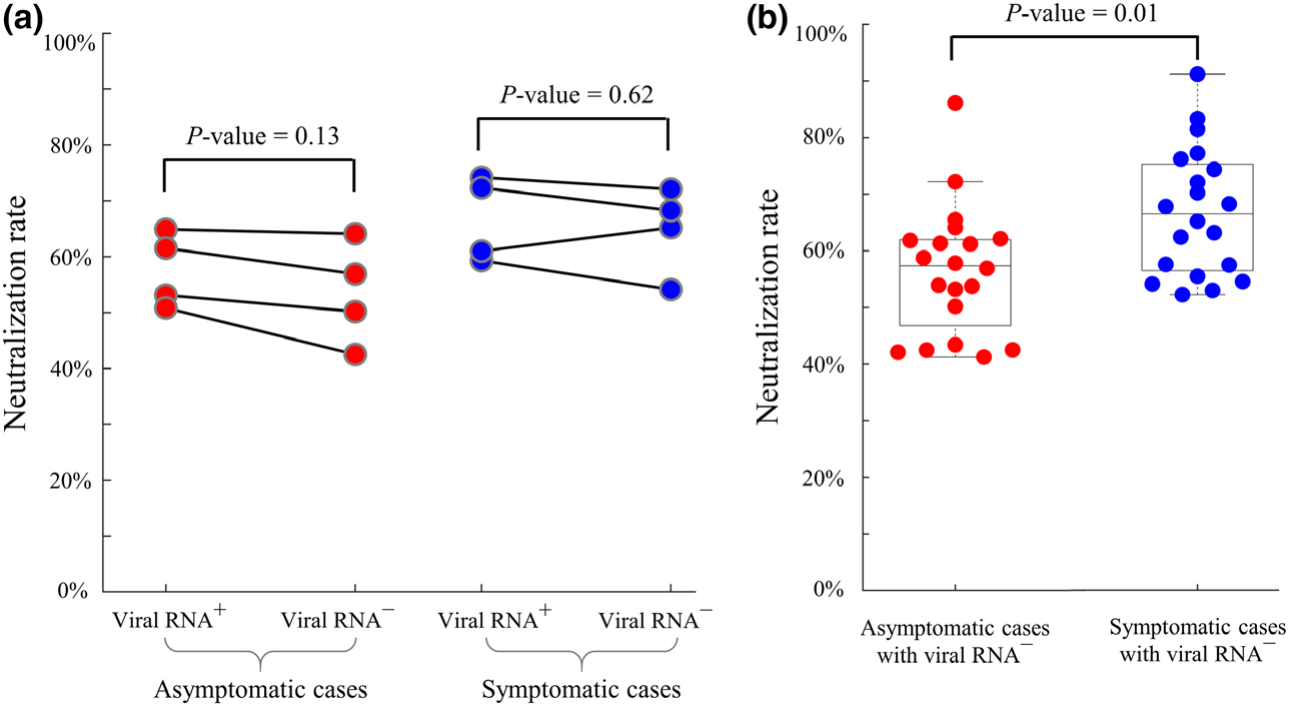
Neutralisation activity of plasmas from asymptomatic and symptomatic patients. **(a)** Temporal changes in neutralisation rates at the transition time points from viral RNA^+^ to viral RNA^−^ in 4 asymptomatic and 4 symptomatic patients. Neutralisation rates were similar within two groups (*P*‐values > 0.05). **(b)** Comparisons of neutralisation rates in 20 recovered asymptomatic and 20 recovered symptomatic patients at the last sampling time points before hospital discharge.

A well‐established pseudovirus‐based neutralisation assay (see Methods) was used to measure the neutralising capacity of plasmas from symptomatic and asymptomatic patients during their hospitalisation. We investigated whether the neutralising capacity would change after the transition from viral RNA^+^ to viral RNA^−^. Neutralisation rates were thus measured using serum samples collected from four asymptomatic and four symptomatic patients with comparable ages and sex proportions. For both groups, neutralisation rates were similar between the sampling time points of viral RNA^+^ and viral RNA^−^ (*P*‐values > 0.05, Figure 5a). We next examined plasma neutralisation rates in 20 recovered asymptomatic patients and 20 recovered symptomatic patients at the last sampling time points before hospital discharge (Figure 5b), and the neutralisation rate was significantly lower in asymptomatic patients (median: 57.4% versus 66.5%, *P*‐value = 0.01).

### Clinical outcomes

By the time of the field hospital closure (10 March 2020), no fatality was reported and 161 (75%) patients who fulfilled discharge criteria were discharged for 14‐day home isolation. The other 53 patients who had positive virus results (*N* = 34), respiratory conditions (*N* = 18) or comorbidities (*N* = 1) were transferred to another hospital for further medical care.

During their hospital stay, 180 (84%) patients experienced virus clearance confirmed by at least two consecutive results of undetectable SARS‐CoV‐2. Virus clearance was observed in similar proportions among the asymptomatic and symptomatic patients (77% versus 85%, *P*‐value = 0.28, Table 3). Furthermore, discharge rates were similar between the asymptomatic and symptomatic patients (77% versus 75%, *P*‐value = 0.83). Among the discharged patients, the length of hospital stay was significantly shorter in asymptomatic than in symptomatic cases (median: 13 versus 18 days, *P*‐value = 0.001).

## Discussion

Early diagnosis and treatment of asymptomatic patients are important for disease control in the fight against SARS‐CoV‐2, but their clinical features are largely unclear. Based on a cohort of 26 asymptomatic and 188 symptomatic patients in a coronavirus field hospital, our study revealed three major findings: (1) asymptomatic patients, mostly young females ≤ 60 years, were observed in approximately 12% of nonseverely ill patients infected with SARS‐CoV‐2; (2) >90% patients experienced IgM/IgG seroconversion at the time of virus clearance, whereas the median time from the first virus‐positive screening to IgG/IgM seroconversion was significantly shorter in asymptomatic than in symptomatic patients; and (3) at the time of virus clearance, asymptomatic patients had lower IgG/IgM titres and plasma neutralisation capacity than symptomatic patients.

We observed asymptomatic cases in approximately 12% of 214 nonseverely ill patients hospitalised in a coronavirus field hospital. The asymptomatic cases were mostly young patients with no SARS‐associated symptoms such as fever and cough (Table 1), suggesting the deficiency of symptom‐based screening to identify asymptomatic cases. Most asymptomatic cases were identified when they received systematic screening for SARS‐CoV‐2 or when their family members or close contacts had been infected with SARS‐CoV‐2. An early survey reported asymptomatic cases in only 1.6% of 56 128 SARS‐CoV‐2 cases as of 11 February 2020,^29^ whereas a higher proportion of asymptomatic cases has been subsequently reported.^12^, ^13^, ^14^, ^15^, ^16^ For instance, (1) 13 (6%) of 216 nonsevere cases in Beijing were asymptomatic^12^; (2) the estimated asymptomatic proportion was 17.9% on the Diamond Princess cruise ship hosting 3711 people^13^; (3) 41 (19%) of 213 COVID‐19 cases recruited from a community facility in South Korea were asymptomatic^16^; and (4) 13 (39%) of 33 COVID‐19 cases from a nursing facility in Illinois were asymptomatic.^15^ Notably, SARS‐CoV‐2 can be efficiently transmitted through active pharyngeal viral shedding even if asymptomatic carriers have no symptoms.^4^ Taken together, accumulated evidence reveals a high proportion of asymptomatic carriers, thereby supporting wide viral screening especially in at‐risk populations. Early identification of asymptomatic cases may be achieved by systematic screening in an enhanced surveillance system.^30^

Unique features of asymptomatic SARS‐CoV‐2 carriers have been reported. First, SARS‐CoV‐2 carriers may exhibit no symptoms during the incubation period and the convalescent phase of the disease.^6^ During their hospitalisation, 54% of our asymptomatic patients were observed to exhibit IgG seroconversion but not IgM seroconversion, but no asymptomatic patient was negative for IgG seroconversion and positive for IgM seroconversion (Table 3). This suggests that many asymptomatic patients may have experienced the early convalescent phase during which IgM decreases and IgG increases. Second, the immune system of asymptomatic patients might have been effectively activated against SARS‐CoV‐2 such that mild symptoms only last for a short period. In a recent study, eight mildly ill patients had a short period (approximately 1 week) of mild symptoms and became asymptomatic after the emergence of IgG/IgM seroconversion within 14 days.^4^ Third, the pathogenicity of SARS‐CoV‐2 is possibly associated with certain viral subtypes or strains,^31^ and further studies are needed to evaluate whether there is a difference in virulence among asymptomatic and symptomatic carriers.

Previous COVID‐19 studies evaluated IgG and IgM seroconversion mostly in symptomatic patients,^4^, ^18^, ^20^, ^26^, ^32^, ^33^ whereas few studies have focused on asymptomatic patients.^34^, ^35^ In a cohort of 173 symptomatic patients, the median time from illness onset to IgM and IgG seroconversion was 12 and 14 days, respectively.^18^ A small‐cohort study of 16 symptomatic patients reported that IgG seroconversion was earlier than IgM seroconversion.^20^ In a cohort of eight symptomatic patients and one asymptomatic patient, IgM/IgG seroconversion was observed within 14 days after hospitalisation.^4^ In our study, we used 448 serum samples to characterise anti‐SARS‐CoV‐2 IgG and IgM antibodies in asymptomatic and symptomatic patients. During their hospitalisation, 85% of the asymptomatic patients experienced IgG/IgM seroconversion, confirming the activation of human immune responses against SARS‐CoV‐2. Moreover, most IgG and IgM seroconversion could be observed at the time of virus clearance (Figure 4a). Our results thus support literature findings that seroconversion correlates with the steady decline in viral loads^4^ and that early immune responses are beneficial to control SARS‐CoV‐2.^36^

We observed significant differences in white blood cells (lymphocytes, eosinophils), red blood cells, IgG/IgM titres and neutralisation activities between asymptomatic and symptomatic patients. In agreement with a recent study of 37 asymptomatic patients and 37 symptomatic patients,^34^ we observed that asymptomatic cases had lower titres of IgG and IgM antibodies (Figure 4b) and neutralisation activities (Figure 5b). Although the exact mechanism remains unclear, there is a general consensus that IgG and IgM levels are associated with disease severity. For instance, IgG/IgM titres in severely ill patients are generally higher than those in nonseverely ill patients.^18^, ^26^, ^37^ Furthermore, serum samples from asymptomatic patients showed higher titres of IgG than IgM antibodies (Figure 3a). It is known that some COVID‐19 patients may have higher levels of IgG than IgM antibodies.^26^ Nevertheless, it is also possible that some asymptomatic patients may have experienced the delayed detection of SARS‐CoV‐2 or hospitalisation such that IgG antibodies could be accumulated to a high level during the prehospital stage. Although the exact interplay between immune responses and disease severity remains unclear, immune responses against SARS‐CoV‐2 might differ between asymptomatic and symptomatic patients. To reveal their immune differences, future studies need to reveal a comprehensive picture of the human immune system against SARS‐CoV‐2 as well as its impact on vaccination.

Although SARS‐CoV‐2 is the main focus of this study, it is worth mentioning the clinical and serological features of other human coronaviruses. Asymptomatic cases in the early survey of laboratory‐confirmed MERS‐CoV cases comprised approximately 12.5%,^38^ which was approximate to the result in our study. IgG and IgM seroconversions were observed in more than half of SARS‐CoV cases 1 week after diagnosis,^39^ but most SARS‐CoV cases experienced IgG and IgM seroconversion within 30 days after symptom onset.^40^, ^41^ Importantly, anti‐SARS‐CoV IgM antibodies remained positive from 30 to 210 days,^41^ though IgG antibodies in SARS‐CoV cases may offer protection for up to 720 days.^42^ A 6‐year follow‐up study reported IgG antibodies in only two (8.6%) of 23 recovered donors at 6 years postinfection of SARS‐CoV,^43^ which suggests the diminishing levels of memory B cells against SARS‐CoV.^44^ Although conserved epitopes are present in the receptor‐binding domains of SARS‐CoV and SARS‐CoV‐2,^45^ recovery from SARS‐CoV infection might not protect patients from SARS‐CoV‐2 infection because of limited cross‐neutralisation.^46^

This study has several limitations. First, serum samples at the first virus‐positive screening were not collected because of the emergent shift in patients during the outbreak. Additionally, the follow‐up data of discharged or transferred patients were unavailable because of the closure of the field hospital. Second, other human coronaviruses were not investigated because SARS‐CoV and MERS‐CoV cases have not been reported in Wuhan in the past decade. Furthermore, four human coronaviruses (HCoV‐HKU1, HCoV‐OC43, HCoV‐NL63 and HCoV‐229E) have a rather low prevalence in China (approximately 0.9% in children, 0.6% in adults; Supplementary table 2), and our medical records indicate no exposure history to other human coronaviruses. Third, our study involved 26 asymptomatic patients, but larger cohorts are required to reveal the prevalence of asymptomatic patients. Moreover, potential associations of IgG/IgM seroconversion with antiviral treatments could not be revealed by our observational study. Future studies also need to characterise the immune system of asymptomatic patients.

## Conclusions

Overall, our study contributes to a better understanding of IgG and IgM antibodies in asymptomatic and symptomatic patients, shedding light on early diagnosis and effective prevention against SARS‐CoV‐2. Although asymptomatic patients are considered healthy before their screening, they carry a highly transmissive source of SARS‐CoV‐2, thereby highlighting the importance of their early diagnosis and treatment. Given its sufficient presence in recovered patients, human convalescent serum with anti‐SARS‐CoV‐2 IgG/IgM antibodies may be used as passive antibody therapy against SARS‐CoV‐2.^47^

## Methods

### Patients and data collection

This retrospective study analysed a cohort of SARS‐CoV‐2 cases hospitalised in the Wuchang field hospital in Wuhan that was temporarily built for treating SARS‐CoV‐2 cases between 5 February and 10 March 2020. At the hospital admission, all patients fulfilled the following inclusion criteria: (1) patients were confirmed with positive results of SARS‐CoV‐2 by viral RNA tests; (2) patients were in good physical condition and had either no symptoms or mild symptoms such as fever or respiratory symptoms; (3) patients had neither mental disorders nor severe dysfunctions of the heart, liver, lung, kidney or brain; and (4) patients had resting pulse oximetry (SpO_2_) > 93% and respiratory rate < 30 breaths min^−1^. To prevent viral transmission, patients without SARS‐CoV‐2 infection were not hospitalised in this field hospital; therefore, they were excluded from our cohort.

During hospitalisation, all patients received the same regimen based on the New Coronavirus Diagnosis and Treatment Guidelines in China (Supplementary method 1). Patients were discharged if they fulfilled all three of the following conditions: (1) at least two consecutive results of undetectable SARS‐CoV‐2 based on nasopharyngeal swabs collected at least 24 h apart; (2) clinical remission of respiratory symptoms and fever for at least three consecutive days; and (3) substantial improvement of both lungs based on computed tomography. Patients who fulfilled discharge criteria were discharged for 14‐day home isolation; other patients were transferred to the Renmin Hospital of Wuhan University because the field hospital was closed on 10 March 2020. Epidemiological, clinical and laboratory results were retrieved from electronic medical records and adapted in the standardised form based on the International Severe Acute Respiratory and Emerging Infection Consortium. All acquired data were cross‐checked by two investigators.

### SARS‐CoV‐2 RNA detection

To meet the urgent request in the field hospital, of SARS‐CoV‐2 RNA was examined by laboratory centres at the Chinese Center for Disease Prevention and Control (from 5 February to 18 February 2020) and the Renmin Hospital of Wuhan University (from 19 February to 10 March 2020). Nasopharyngeal swabs were collected to identify SARS‐CoV‐2 RNA using real‐time RT‐PCR analyses, and the detailed laboratory protocols were reported previously.^48^, ^49^

### Laboratory biomarkers and IgG/IgM antibody tests

Blood samples were collected and stored in 0.05 mm EDTA at 4°C. Assessments of haematologic biomarkers and C‐reactive protein were conducted using a BC‐5390 Hematology Analyzer (Mindray Bio‐Medical Electronics Co., Ltd., Shenzhen, China). Plasma samples were stored at −20°Cbefore IgG/IgM antibody analysis. Quantifications of IgG and IgM antibodies against the nucleocapsid and spike proteins of SARS‐CoV‐2 were measured using an iFlash 3000 Chemiluminescence Immunoassay Analyzer and magnetic particle‐based chemiluminescence immunoassay kits (YHLO Biotech Co., Ltd., Shenzhen, China). This serologic assay with high levels of sensitivity and specificity for detecting SARS‐CoV‐2‐specific IgG and IgM has been validated in previous studies^23^, ^50^, ^51^ and Supplementary figure 6. All procedures were conducted based on the manufacturer's protocols under bio‐safe conditions. Seroconversion was defined as IgG ≥ 10 AU mL^−1^ or IgM ≥ 10 AU mL^−1^. During hospitalisation, serum samples were collected at different time points when blood tests were requested by doctors to monitor disease progression.

### Pseudovirus‐based neutralisation assay

A well‐established pseudovirus‐based neutralisation assay^34^, ^52^ was adapted for measuring plasma neutralisation activity. Briefly, SARS‐CoV‐2 spike‐pseudotyped luciferase‐expressing lentiviruses were used to infect 2 × 10^4^ HEK293T‐hACE2 cells after incubation with diluted sera (1:600)^34^ in 96‐well plates (see reagents and protocols in Supplementary method 2). Experiments were repeated twice. The neutralisation rate of the tested serum was quantified as [RLU_max_ − RLU_serum_]/[RLU_max_ − RLU_background_] × 100% based on the relative light units (RLU) of luciferase activity.

### Statistical analyses

We reported medians and interquartile ranges for continuous variables as well as counts and percentages for categorical variables. Mann–Whitney *U*‐tests and Fisher's exact tests were performed for continuous and categorical variables, respectively. Wilcoxon signed‐rank tests were carried out for matched samples. A common approach called pairwise deletion was applied to handle missing data. Analyses were conducted using MATLAB R2016a.

## Author Contributions

**Chuanhao Jiang:** Conceptualization; Data curation; Formal analysis; Funding acquisition; Investigation; Methodology; Project administration; Resources; Software; Supervision; Validation; Visualization; Writing‐original draft; Writing‐review & editing. **Yali Wang:** Data curation; Formal analysis; Investigation; Resources; Software; Validation; Visualization. **Min Hu:** Conceptualization; Funding acquisition; Project administration; Resources; Software; Supervision; Validation. **Lingjun Wen:** Data curation; Formal analysis; Investigation; Methodology; Resources; Validation. **Chuan Wen:** Formal analysis; Funding acquisition; Resources; Software; Supervision. **Yang Wang:** Data curation; Methodology; Resources. **Weihong Zhu:** Data curation; Investigation; Resources; Supervision. **Shi Tai:** Conceptualization; Data curation; Methodology; Resources. **Zhongbiao Jiang:** Data curation; Formal analysis; Resources; Validation. **Kui Xiao:** Data curation; Investigation; Resources; Validation. **Nuno R. Faria:** Conceptualization; Supervision; Writing‐review & editing. **Erik De Clercq:** Conceptualization; Supervision; Writing‐review & editing. **Junmei Xu:** Formal analysis; Funding acquisition; Project administration; Resources; Supervision; Validation. **Guangdi Li:** Conceptualization; Funding acquisition; Project administration; Software; Supervision; Writing‐review & editing.

## Ethical approval

This retrospective study was performed in accordance with the Helsinki Declaration and was approved by the Ethics Committee of the Second Xiangya Hospital (ID: LYF2020060). Written informed consent was waived for the use of archived medical records and samples.

## Conflict of Interest

The authors declare no conflict of interest.
