# Supplementary material for: Antibody seroconversion in asymptomatic and symptomatic patients infected with severe acute respiratory syndrome coronavirus 2 (SARS‐CoV‐2)
Source: Clin Transl Immunology. 2020 Sep 26;9(9):e1182. doi: 10.1002/cti2.1182 (PMC7519951; doi:10.1002/cti2.1182)
Supplement: Supplementary file 1 [file CTI2-9-e1182-s001.pdf]

Supplementary figures

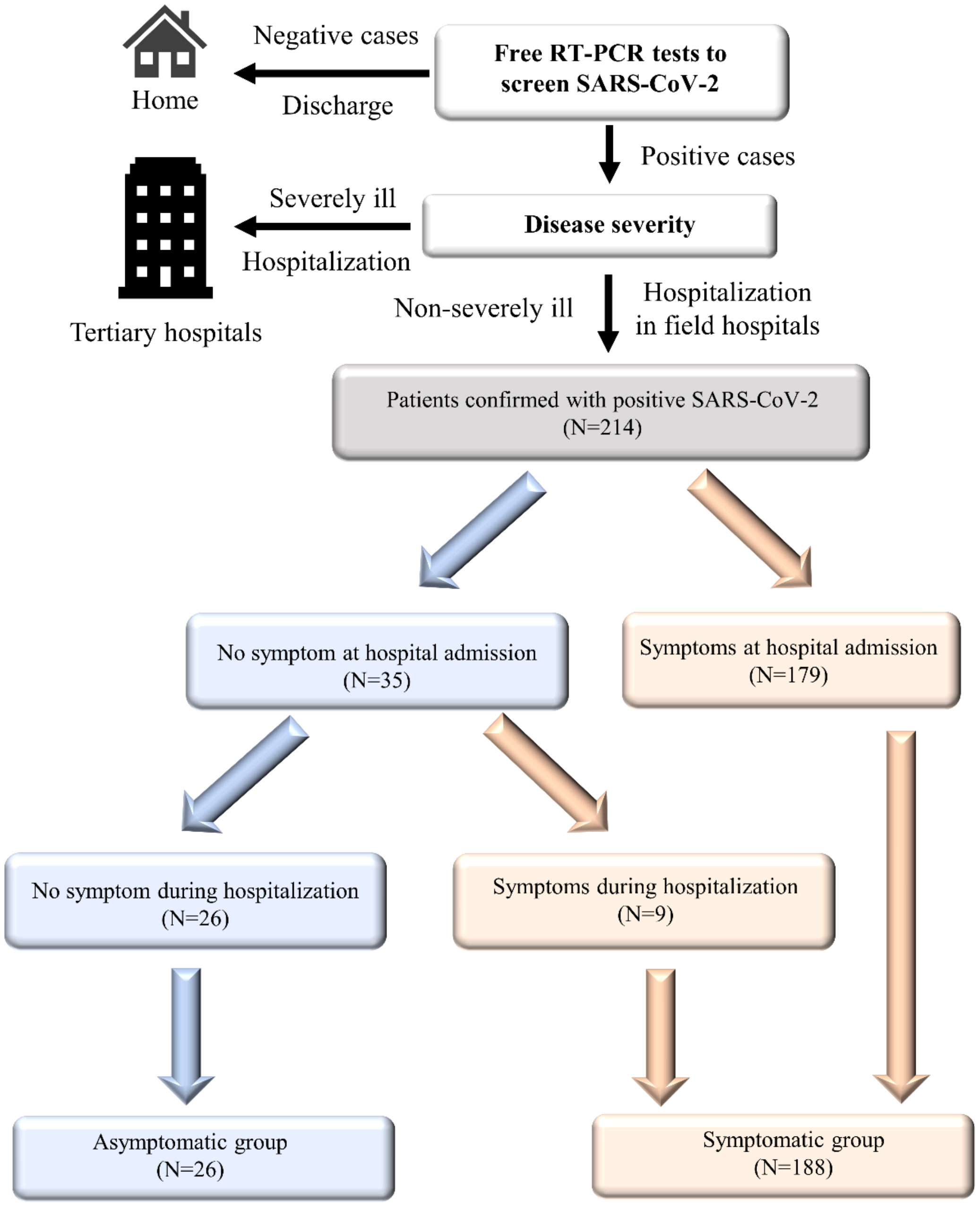

Supplementary figure 1: A flowchart of asymptomatic and symptomatic patients in our study.

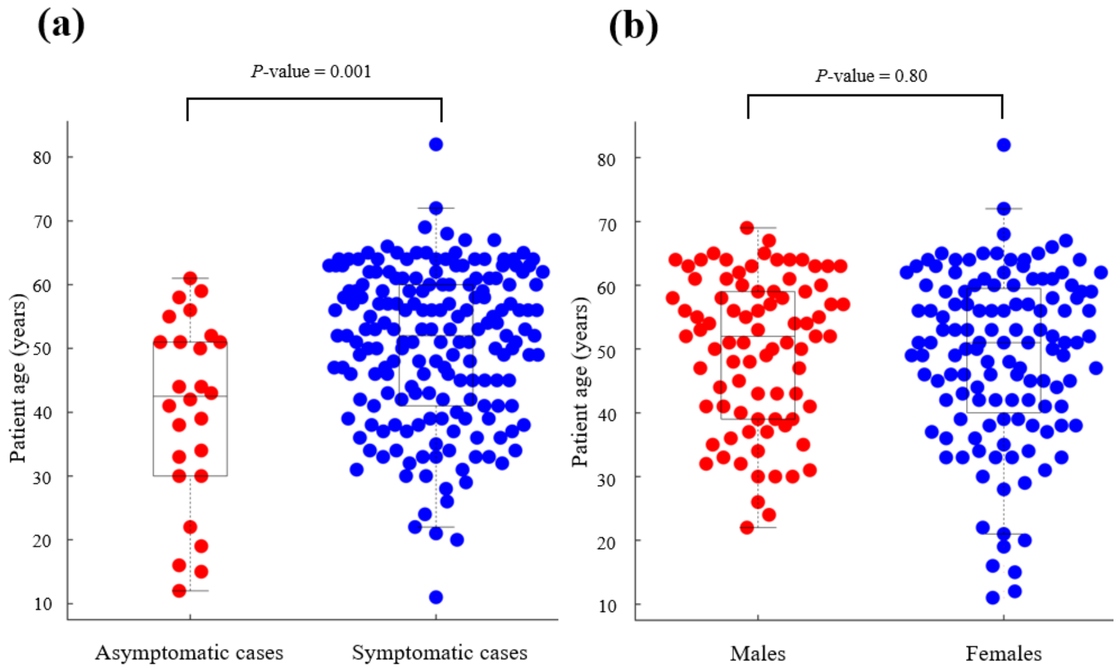

Supplementary figure 2: Age distribution of asymptomatic and symptomatic patients (a), males and females (b).

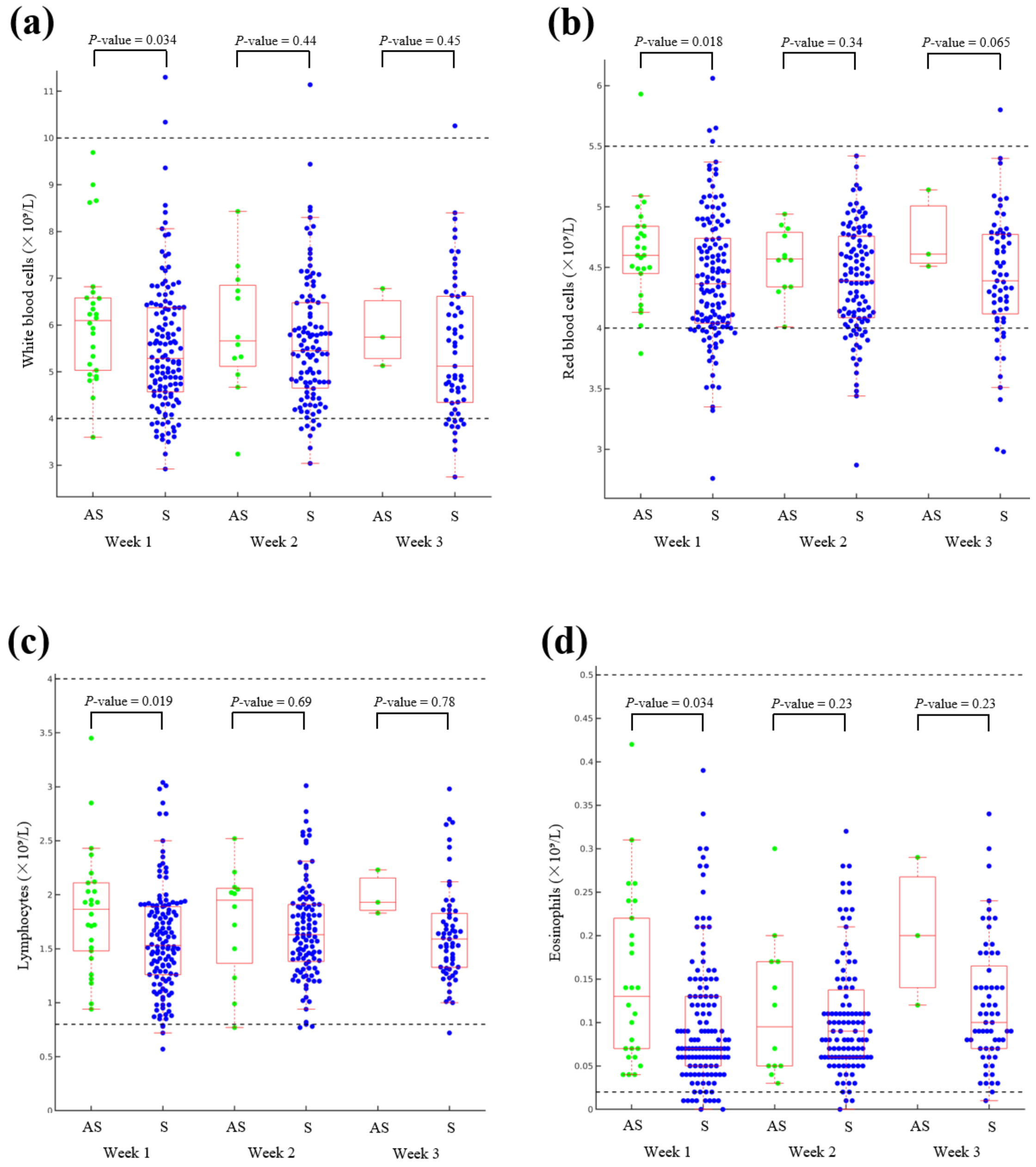

Supplementary figure 3: Longitudinal observations of laboratory biomarkers (white blood cells, red blood cells, lymphocytes, eosinophils) in asymptomatic (AS) and symptomatic (S) patients with COVID-19. Average data were plotted if one patient had two or more blood tests in the same week. For four laboratory biomarkers, statistical significance (*P*-values < 0.05) was consistently observed at week 1.

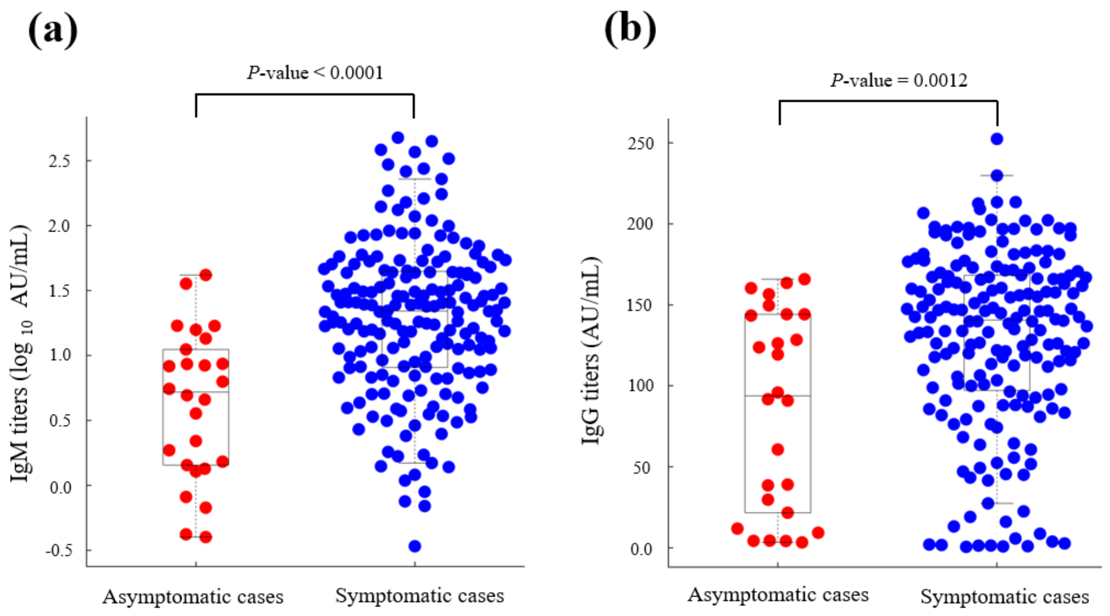

Supplementary figure 4: **Comparisons of IgG and IgM titers in asymptomatic and symptomatic patients.** Boxplots of IgM **(a)** and IgG **(b)** titers in asymptomatic and symptomatic patients. IgG and IgM titers were averaged over all sampling timepoints.

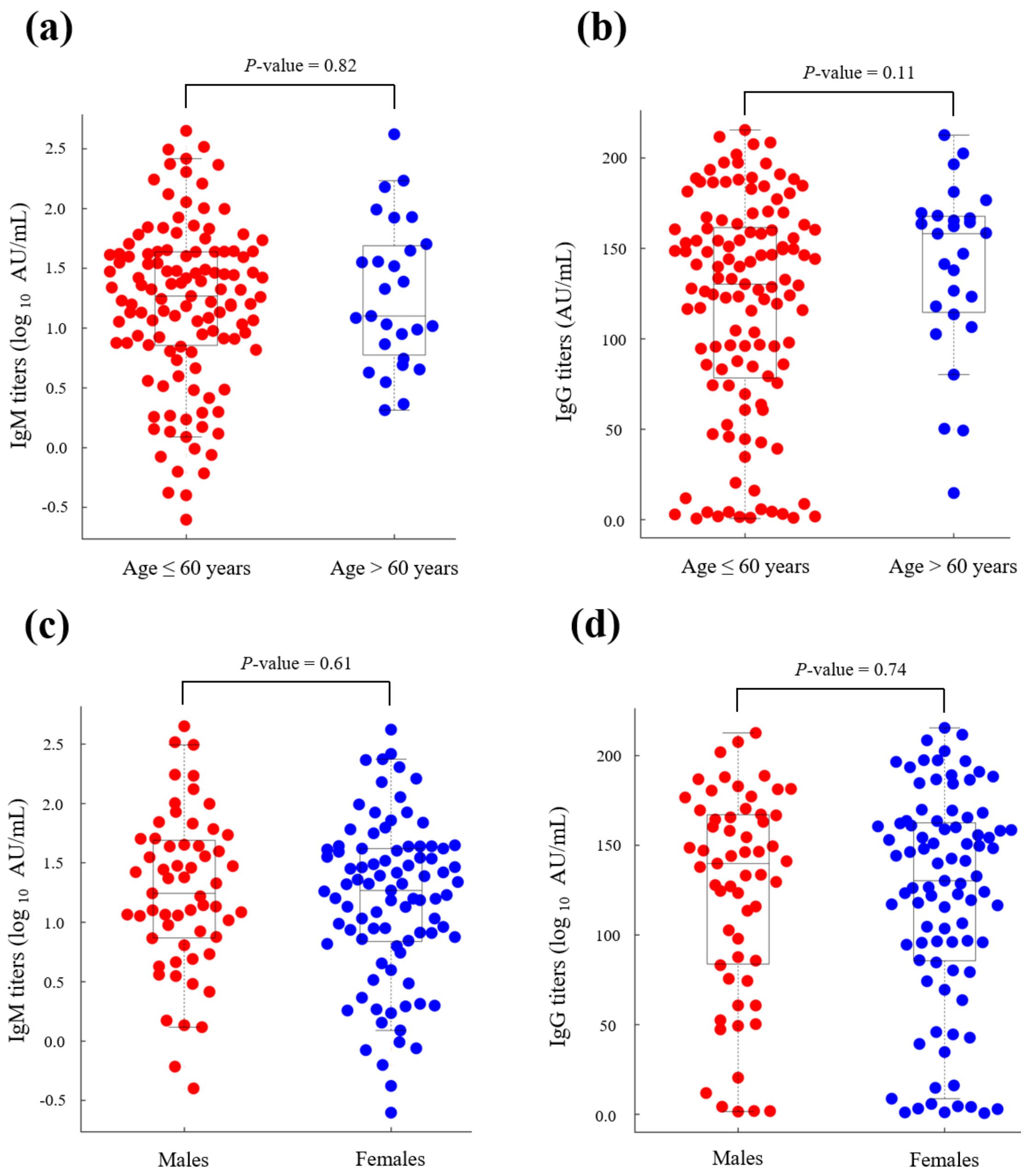

Supplementary figure 5: **Comparisons of IgG and IgM titers in young versus old patients, male versus female patients.** Boxplots of IgM **(a)** and IgG **(b)** titers in young patients (≤ 60 years) and old patients (>60 years). Boxplots of IgM **(c)** and IgG **(d)** titers in male and female patients. No significant differences were identified (*P*-values>0.05).

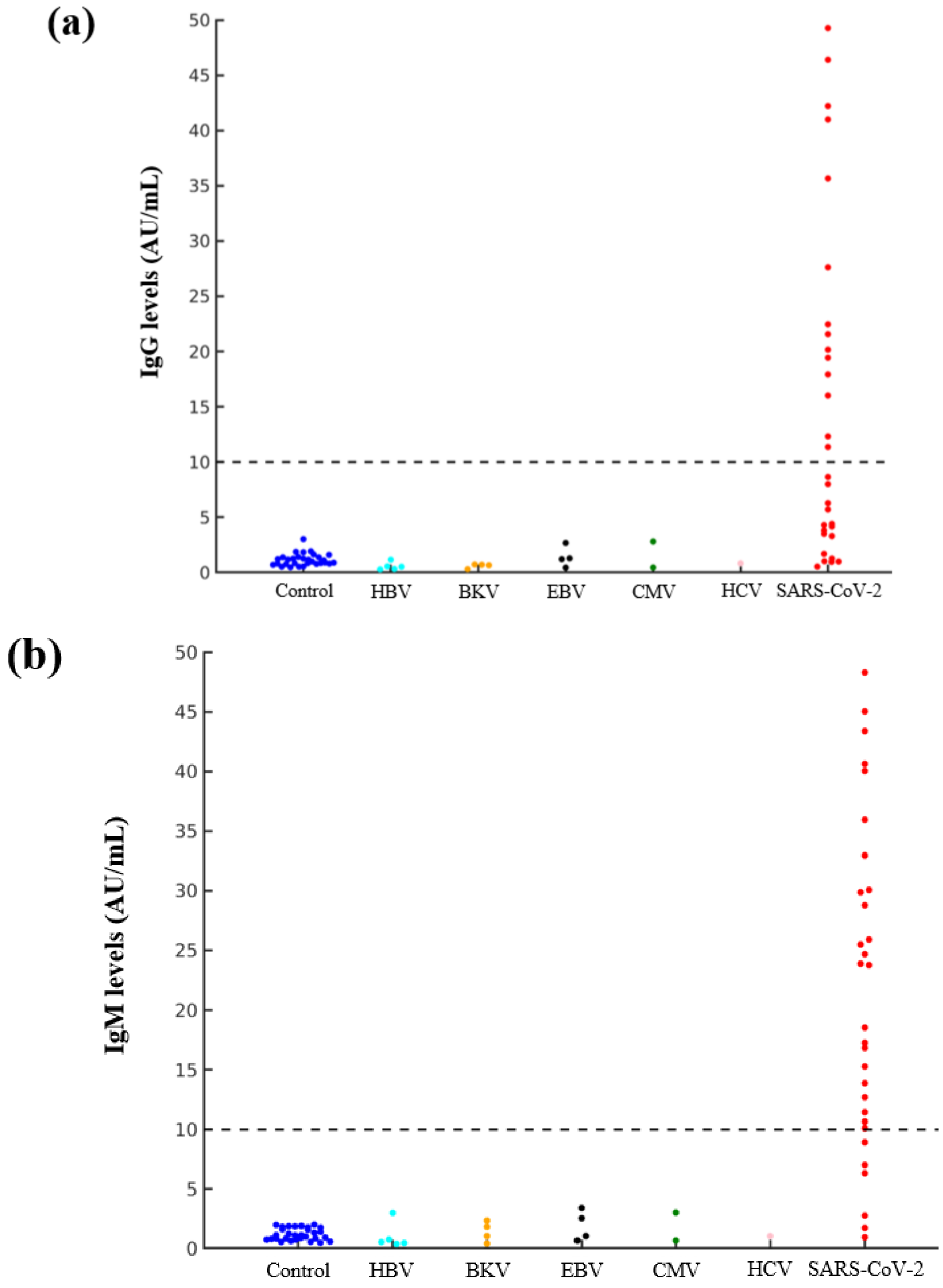

Supplementary figure 6: **Evaluation of IgG and IgM assays in chronic infectious diseases.** Serum samples of healthy subjects (N=30), HBV-infected patients (N=5), BK polyomavirus (N=4), EBV-infected patients (N=4), cytomegalovirus-infected patients (N=2), HCV-infected patients (N=1), and SARS-CoV-2 patients (N=30). All experiments were conducted following standard protocols in the biosafety level 2 laboratory.

Supplementary tables

Supplementary table 1: Normal ranges of laboratory biomarkers in our study

**Laboratory biomarkers**	**Normal range**
White blood cells ($\times 10^9$ L^{-1})	4.00 to 10.00 ($\times$ 10^9 L^{-1})
Neutrophil ($\times 10^9$ L^{-1})	2.00 to 7.00 ($\times$ 10^9 L^{-1})
Lymphocyte ($\times 10^9$ L^{-1})	0.80 to 4.00 ($\times$ 10^9 L^{-1})
Eosinophils ($\times 10^9$ L^{-1})	0.02 to 0.50 ($\times$ 10^9 L^{-1})
Monocyte ($\times 10^9$ L^{-1})	0.12 to 1.20 ($\times$ 10^9 L^{-1})
Basophils ($\times 10^9$ L^{-1})	0.00 to 0.10 ($\times$ 10^9 L^{-1})
Red blood cells ($\times 10^{12}$ L^{-1})	4.00 to 5.50 ($\times$ 10^{12} L^{-1})
Hemoglobin ($g.L^{-1}$)	120 to 160 $g.L^{-1}$
Platelet ($\times 10^9$ L^{-1})	100 to 300 ($\times$ 10^9 L^{-1})
C-reactive protein ($mg.L^{-1}$)	0.00 to 4.00 ($mg.L^{-1}$)

Supplementary table 2: Prevalence of HCoV-HKU1, HCoV-OC43, HCoV-NL63, and HCoV-229E in China

Samples	Age classes	HCoV-OC43	HcoV-229E	HcoV-HKU1	HcoV-NL63	Ref.
Nasopharyngeal samples (N=8275)	Children (≤15y)	12 (0.15%)	5 (0.06%)	4 (0.05%)	2 (0.02%)	[1]
	Adults (>15y)	36 (0.44%)	7 (0.08%)	7 (0.08%)	4 (0.05%)	
Throat and nasal swab specimens (N=13048)	Children (≤15y)	153 (1.17%)	34 (0.26%)	13 (0.10%)	22 (0.17%)	[2]
	Adults (>15y)	24 (0.18%)	15 (0.11%)	10 (0.08%)	22 (0.17%)	
Nasopharyngeal swabs (N=3298)	Children (≤6y)	36 (1.09%)	2 (0.06%)	34 (1.03%)	6 (0.18%)	[3]
Nasal and throat swabs (N=8396)	Adults (>15y)	50 (0.60%)	15 (0.18%)	14 (0.17%)	8 (0.10%)	[4]
						Overall prevalence
Total (N=33017)	Children (≤15y)	201 (0.61%)	41 (0.12%)	51 (0.15%)	30 (0.09%)	323 (0.9%)
	Adults (>15y)	110 (0.33%)	37 (0.11%)	31 (0.09%)	34 (0.10%)	212 (0.6%)

Supplementary Method 1

Description of the recommended treatment in the field hospital

Based on the New Coronavirus Diagnosis and Treatment Guidelines (version 5 and 6) in China, all patients received the same regimen during their hospitalization in the Wuchang field hospital. The recommended regimen includes (i) umifenovir, (ii) Lianhua-Qingwen capsule [5], and (iii) traditional Chinese medicines (radix bupleuri 20 g, radix scutellariae 10 g, rhizoma pinelliae preparata 10 g, codonopsis pilosula 15 g, fructus trichosanthis 10 g, semen arecae 10 g, fructus tsaoko 10 g, cortex magnoliae officinalis 15 g, rhizoma anemarrhenae 10 g, paeonia lactiflora 10 g, raw radix glycyrrhizae 10 g, pericarpium citri reticulatae 10 g, polygonum cuspidatum 10 g).

Before the submission of this study, anti-SARS-CoV-2 activities of traditional Chinese medicines had not been proved by any randomized double-blind clinical trial. Their clinical use and their impacts on immunological responses remain unclear. Since all asymptomatic and symptomatic patients received the same regimen in our study, our major findings are unlikely affected, while future studies need to clarify associations of immune responses and the recommended regimen.

Supplementary Method 2

Description of pseudovirus-based neutralization assay

A well-established pseudovirus-based neutralization assay [6, 7] was adapted for measuring the plasma neutralization activity. Briefly, HEK293T-hACE2 cells (2×10^4 cells/well) were cultured in a 96-well plate for 12 hours before infection. After heat-inactivation at 56°C for 60 minutes, diluted serum samples (1:600) [7] were mixed with the equal volumes (50 µL) of SARS-CoV-2 spike-pseudotyped luciferase-expressing lentiviruses and the mixtures were incubated at 37°C for one hour. The sera-pseudovirus mixtures and polybrene (5 µg/mL per well, Sigma Aldrich) were added to HEK293T-hACE2 cells in a 96-well plate and subsequently incubated at 37°C for 48 hours. The relative light units (RLU) of luciferase activity were measured using the Luc-Pair™ Firefly Luciferase HS Assay Kit (Promega) and EnSpire® Multimode Plate Reader (PerkinElmer) according to the manufacturer's protocols. Experiments were repeated twice.

Similar to the definition in [7], the neutralization rate (%) was quantified as:

$$\frac{RLU_{max} - RLU_{serum}}{RLU_{max} - RLU_{background}} \times 100\%$$

Where RLU_{max} represents the maximum infection without any serum and it is measured by the average RLU signal of positive-control wells with pseudoviruses plus HEK293T-hACE2 cells; RLU_{serum} is the RLU signal of experimental wells with patient serum plus pseudoviruses and HEK293T-ACE2 cells; $RLU_{background}$ is the background RLU signal averaged from the "pseudovirus only" and "pseudovirus+HEK293T" wells. The higher the neutralization rate, the stronger the inhibition effect of patient serum.

References

1. Yip CC, Lam CS, Luk HK, Wong EY, Lee RA, So LY et al. A six-year descriptive epidemiological study of human coronavirus infections in hospitalized patients in Hong Kong. Virol Sin. 2016;31(1):41-8. doi:10.1007/s12250-016-3714-8.

2. Zhang SF, Tuo JL, Huang XB, Zhu X, Zhang DM, Zhou K et al. Epidemiology characteristics of human coronaviruses in patients with respiratory infection symptoms and phylogenetic analysis of HCoV-OC43 during 2010-2015 in Guangzhou. PLoS One. 2018;13(1):e0191789. doi:10.1371/journal.pone.0191789.

3. Liu P, Shi L, Zhang W, He J, Liu C, Zhao C et al. Prevalence and genetic diversity analysis of human coronaviruses among cross-border children. Virol J. 2017;14(1):230. doi:10.1186/s12985-017-0896-0.

4. Ren L, Gonzalez R, Xu J, Xiao Y, Li Y, Zhou H et al. Prevalence of human coronaviruses in adults with acute respiratory tract infections in Beijing, China. J Med Virol. 2011;83(2):291-7. doi:10.1002/jmv.21956.

5. Jia W, Wang C, Wang Y, Pan G, Jiang M, Li Z et al. Qualitative and quantitative analysis of the major constituents in Chinese medical preparation Lianhua-Qingwen capsule by UPLC-DAD-QTOF-MS. ScientificWorldJournal. 2015;2015:731765. doi:10.1155/2015/731765.

6. Crawford KHD, Eguia R, Dingens AS, Loes AN, Malone KD, Wolf CR et al. Protocol and Reagents for Pseudotyping Lentiviral Particles with SARS-CoV-2 Spike Protein for Neutralization Assays. Viruses. 2020;12(5):513. doi:10.3390/v12050513.

7. Long QX, Tang XJ, Shi QL, Li Q, Deng HJ, Yuan J et al. Clinical and immunological assessment of asymptomatic SARS-CoV-2 infections. Nat Med. 2020;26(8):1200-4. doi:10.1038/s41591-020-0965-6.
